# Enhancement of Thermal Behaviour of Flax with a Ramie Fibre-Reinforced Polymer Composite

**DOI:** 10.3390/polym15020350

**Published:** 2023-01-09

**Authors:** Durvasulu Rajesh, Nagarajan Lenin, Robert Cep, Palanivel Anand, Muniyandy Elangovan

**Affiliations:** 1 Department of Mechanical Engineering, Vel Tech Rangarajan Dr. Sagunthala R&D Institute of Science and Technology, Avadi 600062, India; 2 Department of Machining, Assembly and Engineering Metrology, Faculty of Mechanical Engineering, VSB-Technical University of Ostrava, 17. Listopadu 2172/15, 70800 Ostrava, Czech Republic; 3 Department of R&D, Bond Marine Consultancy, London EC1V 2NX, UK

**Keywords:** flax, ramie, heat deflection temperature, hybrid green composites, thermogravimetric analysis, thermal expansion, thermal properties

## Abstract

Plant-derived fibres, called lignocellulosic fibres, are a natural alternative to synthetic fibres in polymer composite reinforcement. Utilizing renewable resources, such as fibre-reinforced polymeric composites made from plant and animal sources, has become a crucial design requirement for developing and producing parts for all industrial goods. Natural-fibre-based composites are used for door panels, trays, glove boxes, etc. This study involves developing and thermal analysing a flax fibre reinforced with phenol–formaldehyde resin hybridization with ramie fibre by way of a vacuum infusion process. As per ASTM Standard, eight different sequences were fabricated and thermally characterized. In the present study, three stages of weight loss (%) are shown by the thermogravimetric analysis (TGA). The sample loses less weight during the first stage, more during the second, and more during the third. The sample’s overall maximum temperature was recorded at 630 °C. It was discovered that sample D (80.1 °C) had the highest heat deflection temperature, and sample B had the lowest (86.0 °C). Sample C had a low thermal expansion coefficient, while sample G had a high thermal expansion coefficient. Sample E had the highest thermal conductivity, measured at 0.213 W/mK, whereas sample A had the lowest conductivity, at 0.182 W/mK. From the present study, it was found that sample H had better thermal characteristics. The result of the present investigation would generate thermal data regarding hybrid ramie and flax composites, which would be helpful for researchers and practitioners involved in the field of biocomposites.

## 1. Introduction

Composites are materials made from two or more distinct components with significantly different physical or chemical properties [1,2]. These materials have a wide range of applications, including in the aerospace, automotive, and construction industries [3,4]. The scope of research on composites is broad and includes studies on their mechanical properties, manufacturing and machining processes, and potential for use in various applications [5,6].

One of the current trends in the polymer industry is natural fibre composites. Natural fibre composites have several benefits over typical polymer composites, including being made from renewable materials (which reduces the demand on petroleum resources), being biodegradable, being less abrasive to processing equipment, and being more affordable [7]. Such composites use fibre reinforcements made from natural resources. Hemp, flax, sisal, jute, cotton, etc., are a few of the natural fibres used most frequently [8]. Biocomposite materials are becoming increasingly popular nowadays since they are biodegradable, more environmentally friendly than traditional petroleum-based composites, and cost-effective because at least one of the raw elements utilised in the composite is derived from renewable natural resources. In comparison to conventional composites, this composite offers less human risk and safer working conditions during manufacture. However, environmental factors, soil quality, and plant husbandry compromise the natural fibre’s uniformity in the natural fibre composite. Moreover, when making composites, the hydrophilic nature reduces compatibility with the hydrophobic polymer matrix. In addition, natural fibres have poor dimensional stability due to their hydrophilic nature since they expand when exposed to water.

On the other hand, natural fibres have many advantages, such as their organic makeup, low price, and low density. As a result, they can be utilised in place of artificial fibres. Natural fibres can take various physical forms depending on fibre isolation but are often not single filaments like most man-made fibres [9]. The maximum temperature at which a natural fibre decomposes is its thermal stability [10]. It is well known that different parts of natural fibres, such as hemicellulose, lignin, etc., degrade at different temperatures, which causes the fibre to decompose [11] completely. Therefore, the degree of isolation has a significant impact on tensile strength. Natural fibres are used to make a variety of yarns, mats, textiles, and nonwovens. The natural fibre microstructure is exceedingly complex since it consists of various hierarchical microstructures. The single or elementary fibres are made of a microfibrillar cellulose phase and a matrix phase that is primarily made of hemicellulose and lignin. They have a diameter of around 10 to 20 µm. The fibre’s mechanical strength comes from the cellulosic fibrils, composed of 30 to 100 cellulose molecules in extended chain conformations and have a diameter of around 10 nm [12]. Natural-fibre-based composites are utilised in the automobile sector for interior parts such as door panels, dashboards, seatbacks, packaging trays, and glove boxes, and in the engineering market.

**Figure 1:**
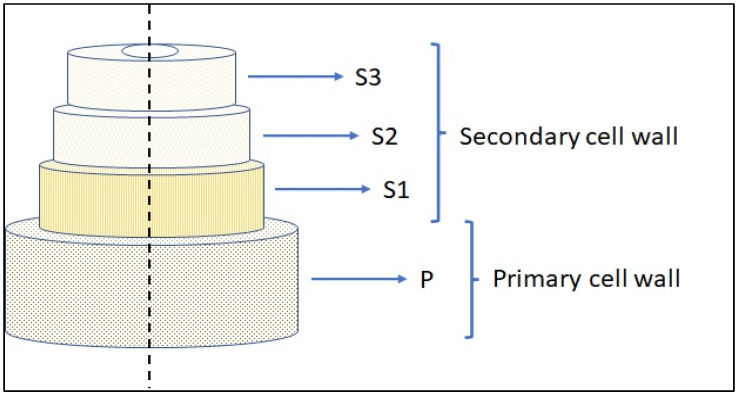
Pictorial representation of fibre structure.

The oilseed flax stalk can produce pure flax fibre; doing so is time-consuming and expensive. Hence, most flax fibre contains shives. Flax fibre has a low lignin level of about 5%, but flax shives are woody materials with a lignin content of about 24% [13]. However, cellulose levels in both shive and flax fibre are high. Natural fibres are primarily composed of cellulose and hemicelluloses, which include many hydroxyl groups. Figure 1 shows the pictorial representation of a typical fibre structure. It consists of two cell walls, namely the primary cell wall and the secondary cell wall. The primary cell wall constitutes an outer wall of the fibre, and the secondary cell wall consists of the inner part of the fibre [14].

Ramie fibres are widely available because they may be harvested with great productivity three times per year. Therefore, only the best fibre in the ramie plant’s outer culm can be used [15]. A ramie fibre’s length typically spans from 60 to 500 mm, while its diameter typically falls between 20 and 35 m. Ramie fibre contains 65–75% cellulose and 1–2% lignin compared to wood, which has cellulose and lignin of 40–50% and 15–35%, respectively. This is one of the strongest of all plant fibres [16]. The ramie fibre peeled from the ramie culm comprises bundles of numerous ramie fibres bound together. To avoid fibre damage, these bundles can either be treated to separate to the necessary diameter or used immediately without separation [17]. Bio-based polyurethane resin is derived from tannin and black liquor when impregnated into ramie fibre to improve their mechanical and thermal properties, enhancing the ramie fibre’s value in industrial application as a functional material [18,19].

Chemical structure, including the degree of polymerization, cellulose concentration, crystallinity, and orientation, significantly impacts the quality of natural plant fibres, origin, plant quality, age, location, weather, and processing [20,21]. Due to the high curing temperatures used in developing thermoset resins or too-high extrusion temperatures with thermoplastic resins, fibre thermal characteristics are of utmost importance. Since natural cellulose fibres consist of different organic components (cellulose, hemicellulose, lignin, etc.), they undergo a wide range of chemical and physical changes when subjected to high temperatures [22]. Tezara et al. [23] investigated the hybridization effect of a jute–ramie-reinforced epoxy composite. The selection of materials and proper stacking sequence predominantly affect the mechanical properties and water absorption behaviour. Hemicellulose and cellulose have been proven to break down much more quickly than lignin when exposed to heat. Using thermogravimetric analysis (TGA), it was discovered that lignin degrades between 280 and 300 °C, and hemicellulose breaks down the natural fibres between 220 °C and 280 °C. The lignin, which is primarily composed of hydrocarbon, aids in the production of char and protects the fibres from further thermal deterioration [24]. Sridhar et al. [25] investigated the thermal stability of jute fibres; they discovered that the depolymerization of the fibres caused a 60% reduction in tensile strength at 300 °C under vacuum conditions for two hours. In addition, on the other hand, they investigated the thermal deterioration of wood/polymer composites at temperatures between 220 °C and 260 °C. Even if heat deterioration caused the tensile strength and modulus to decline, it was reported that the thermal degradation results in porous polymer products, which may result in low density and affect the mechanical properties adversely [26].

Individual thermal analyses of both fibres are reported in various literature. A hybrid composite, which is the combination of flax and ramie fibres, has not been studied until now. The present investigation shows the thermal behaviour of flax with ramie fibres reinforced with phenol–formaldehyde resin. It tested for thermal properties such as thermogravimetric analysis, thermal conductivity, the coefficient of thermal expansion, and heat deflection temperature. Morphological analysis was conducted under a scanning electron microscope to examine the failed surface of the composite.

## 2. Materials and Methods

### 2.1. Flax and Ramie Fibres

**Figure 2:**
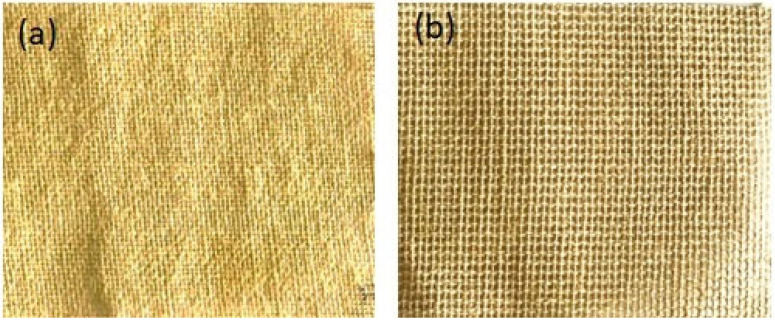
Photographs of (**a**) flax fibre and (**b**) ramie fibre.

Flax and ramie fibres were procured from Go Green Products Pvt. Ltd. (Chennai, India). The photograph of both the fibres is shown in Figure 2a,b. Both fibres offer bidirectional features. Both flax and ramie fibres are derived from flowering plants, named *Linum Usitatissimum* and *Urticaceae*. When derived from the plant, flax fibre is slightly more durable than cotton fibre. It is primarily used in the textile industry in Western nations since it is a strong and durable fibre. The advantages of flax fibre include its density, renewable status, and lower risk compared to glass fibres, and products created from it do not typically lose their shape. On the other hand, ramie fibre is among the most rigid fibres and keeps its strength when wet. Table 1 provides the material characteristics of both fibres. We can observe that high strength was observed in ramie fibre compared to flax fibre.

### 2.2. Phenol–Formaldehyde Resin

Phenol–formaldehyde, also known as phenolic resin, outperforms other resins regarding surface smoothness, strength, affordability, and fire resistance. By reacting phenol and formaldehyde, these resins are artificial polymers [27]. First, in a 12.5:1 ratio, the resin and hardener are combined. Next, the catalyst mixture is applied to the resin and hardener for 30 min while being continually agitated for 5 min. Curing and postcuring of the composite are essential to obtain suitable properties. Finally, the fabricated composite laminate is cured in a room-temperature environment for 24 h. The Hyderabad-based ABR Organics Limited Telangana provided hardener and phenol–formaldehyde resins.

### 2.3. Vacuum-Bagging Resin-Infusion Process

A conventional hand layup involves placing the reinforcement into a mould and manually wetting them with brushes, rollers, or other tools. The laminate can be improved by sucking extra resin through the vacuum-bagging resin-infusion process. During the vacuum-infusion process, a laminate is pressed under a vacuum, which forces the resin within [28]. Dry materials are inserted into the mould after it has been gel-coated, and then a perforated release film is placed on top of the dry reinforcement. Before applying the resin, a vacuum is created, and the dry components are crushed. Once the dry components are thoroughly vacuumed, the resin is infused into them using properly placed tubing. High resin-to-glass ratios are possible with vacuum infusion, and the laminate mechanical characteristics are superior [29,30]. One of the significant advantages of the vacuum-bagging resin-infusion process is that it significantly enhances the fibre-to-resin ratio.

### 2.4. Fabrication Procedure

**Figure 3:**
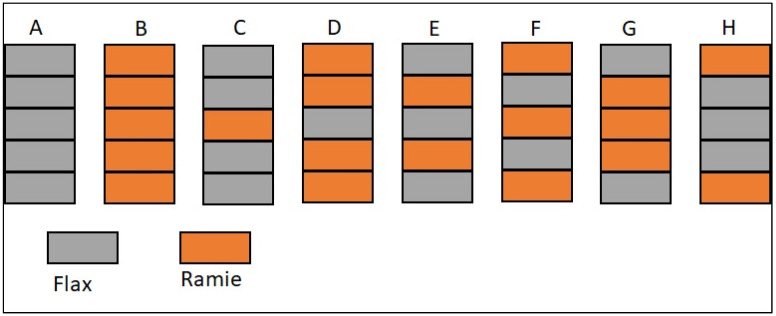
Stacking sequences of the hybrid composites.

Vacuum infusion is used to prepare composite specimen fabrication. It is one of the most economically viable manufacturing methods among the different moulding methods. Initially, a mould should be designed with a size of 300 × 300 x t mm^3^, cleaned, and have a released agent applied. A mixture of phenol–formaldehyde resin and hardener in a 12.5:1 ratio was utilised as a matrix. For sample specimen removal, the bottom of the mould is coated with a releasing agent. After the coating has dried, the initial layer of fibre is left on the coated surface. The resin is then forced through the laminate using vacuum pressure as the other four layers of fibres are kept in place one after the other. Once a complete vacuum has been produced, the tube is carefully positioned to draw resin into the laminate. This experimental work included eight various stacking sequences, as indicated in Figure 3. At room temperature, it is allowed to cure for 24 h. Next, the laminate is removed from the vacuum setup; once the curing process is finished, it cuts as per the ASTM specifications. Table 2 shows the stacking sequence of various samples, the volume of the laminates, and the flax and resin fibre thicknesses.

### 2.5. Testing of Composites

#### 2.5.1. Thermogravimetric Analysis (TGA)

TGA, in accordance with ASTM Standard E 1131, was used to assess the thermal degradation of the raw polymer, fibres, and composites [31]. TGA examines how a material’s physical and chemical properties change (with a constant heating rate) as temperature rises. Samples of approximately 5 mg in weight were heated at a rate of 50 °C per minute in a nitrogen atmosphere using a Pyris 1 instrument from Perkin-Elmer, Italy, and the accompanying weight loss was recorded.

#### 2.5.2. Heat Deflection Temperature

Heat deflection temperature is the temperature at which a typical test bar bends a specified distance under load. It is applied to assess momentary heat resistance. It distinguishes between materials that retain their stiffness throughout a narrow temperature range and those that lose it at higher temperatures while supporting low loads. Samples measuring 127 mm, 12.7 mm, and 3 mm were placed beneath the deflection measurement apparatus. Each specimen was subjected to a load of 1.80 MPa. The temperature was increased by 2 °C per minute in a bath of silicone oil until the samples deflected by 0.25 mm, as specified by ASTM D 648.

#### 2.5.3. Coefficient of Linear Thermal Expansion

The rate of a material’s expansion in relation to temperature is calculated using linear thermal expansion. This test can be used to check for potential thermal stress failure as well as for design purposes. A successful application may need knowledge of two materials’ relative expansion/contraction properties. There was adherence to ASTM Standard D 696 [32]. This analysis made use of a dilatometer. At room temperature, samples measuring 127 mm, 12.7 mm, and 3 mm were placed in the dilatometer, and the height gauge was set up and zeroed. The samples were tested between 30 and 70 °C with the instrument submerged in a temperature bath.

#### 2.5.4. Thermal Conductivity

An apparatus with a guarded hot plate was used for testing. On either side of the primary heater, two identical samples were positioned, each having a diameter of 50 mm and a thickness of 10 mm. The temperature of both the guard heaters and the main heater was maintained at 55 °C. The temperatures of both auxiliary heaters were kept lower. The primary heater’s lateral heat transfer was reduced to a minimum by the guard heaters. Thermocouples were used to measure the temperatures at each surface. The power provided to the primary heater was equivalent to the heat passed through the sample. Thermal equilibrium was reached when both the temperature and voltage readings were constant. The ASTM Standard E 1530 was adhered to for the abovementioned test [33]. The thermal conductivity of phenol–formaldehyde resin was 0.25 W/mK, and its density was 1300 kg/m^3^ [34].

#### 2.5.5. Morphological Analysis

SEMs are electron microscopes with the ability to create high-resolution, three-dimensional images of a sample’s surface. The SEM’s resolution is decided mainly by the size of the electron beam’s spot as it hits the material. A sample’s electrons are distributed in an inelastic manner. A scintillator–photomultiplier then picks them up for further analysis. The morphology of the polymer composites can be studied in detail using SEMs. During “scanning”, the electron beam interacts with the composite’s surface, producing secondary electrons. The screen shows a picture, with contrasting colours representing the composite’s exterior. Micrographs taken with an SEM are commonly used in the study of composite morphology, as they reveal details such as dispersion, interfacial bonding, fibre pull-out, filler particle size, etc. Experimental examination of composites benefits significantly from the information disclosed by these SEM pictures of the samples’ underlying mechanics. For example, pulled-out fibres in the SEM photos indicate poor adhesion between fibre and matrix.

In this work, specimens were studied with a JOEL JSM 6490 SEM. Before the SEM examination, a layer of gold (10 nm thick) was sputtered onto all samples. The carbon adhesive tabs were double-sided for electrical conductivity and were used to attach each specimen to the microscope’s aluminium holder. The accelerating voltage used ranged between 5 and 30 kV.

## 3. Results and Discussion

### 3.1. Thermogravimetric Analysis (TGA)

**Figure 4:**
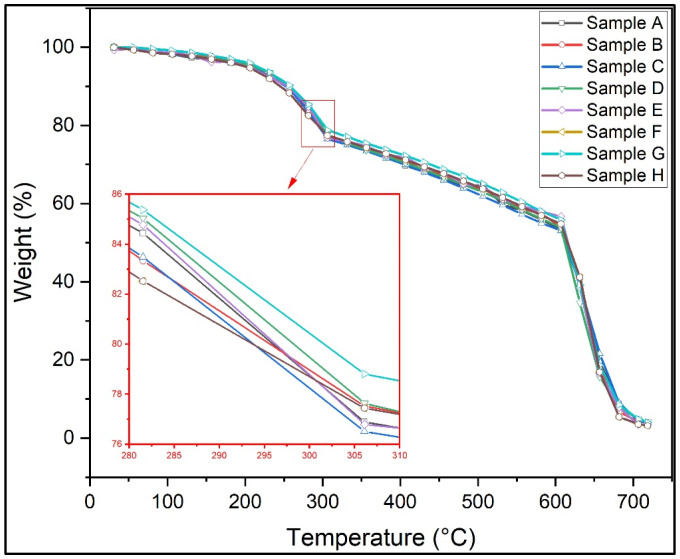
Thermogravimetric analysis of the test samples.

**Figure 5:**
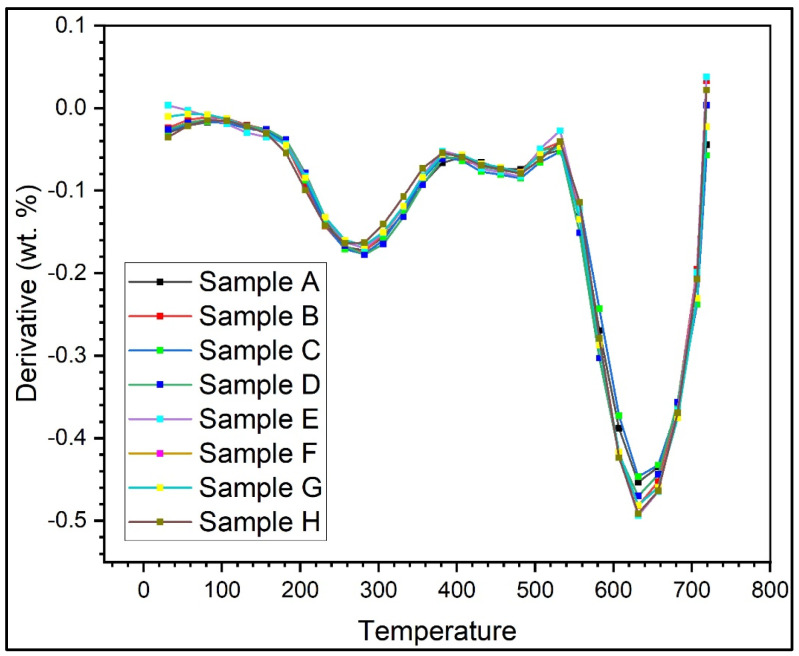
Thermogravimetric analysis graph between the derivative of weight and temperature.

**Figure 6:**
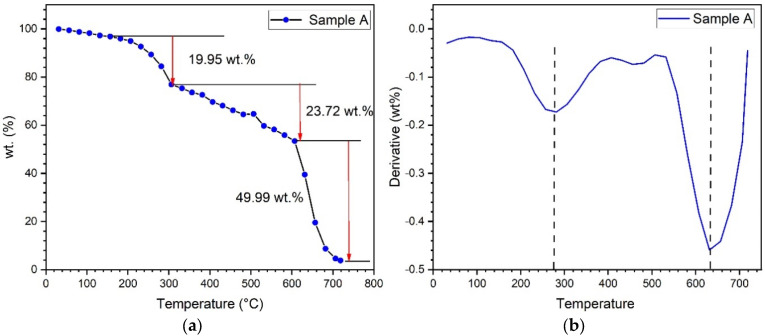
(**a**) TGA graph showing three regions; (**b**) graph representing maximum temperature.

**Figure 7:**
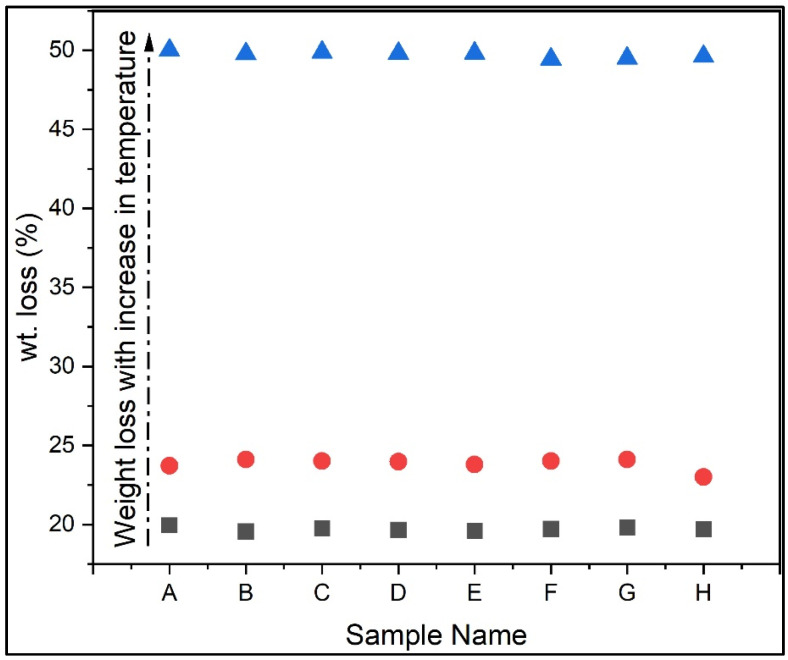
Graph showing an increase in weight loss (%) with an increase in temperature.

TGA evaluated the moisture absorption, thermal stability, and fibre degradation. Figure 4 shows the thermogravimetric analysis of all of the test samples, and the plot shows a decrease in weight percentage in all samples. Figure 5 shows the thermogravimetric analysis graph between the derivative of weight and temperature. The thermodegradation of the samples can be divided into three temperature regions. The first region (I) was a temperature range between 30 °C and 270 °C, followed by a temperature range for the second region (II) of between 270 °C and 630 °C, and, finally, the third region (III) was between 630 °C and 720 °C. Figure 6a shows the representation of the three regions and their weight loss (%) at various stages. In the present study, from Figure 6a, it was observed that the curve initially had a slight decrease in region I. This slight decrease in the region was mainly due to the evaporation of formaldehyde, water, phenol, and volatile substances [35]. This region was associated with a small amount of mass loss. The second region constitutes an approximate linear region in the graph. This region consists of the cellulose degradation of fibres and, from the condensation, reactions between phenolic OH and methylene and between two hydroxyl functional groups found in the resin [36]. In the third region, there was a sudden increase in the weight loss percentage. Almost significant weight loss was observed in this region. The degradation was due to the degradation of fibre and the cross-linking of methylene with carbon–hydrogen cross-links causing hydrogen elimination in the resin. Even more so, the methylene group would react with the water and hydrogen generated, yielding carbon monoxide and methane, respectively. From Figure 6b, it was found that higher weight loss was observed in sample G, and the most negligible weight loss was observed in sample D. Figure 6 shows the graph between the derivative of weight loss percentage and temperature. Figure 7 shows the maximum temperature in the samples as all the samples show similar behaviour in the graph. The maximum temperature was found to be 630 °C. Figure 7 shows the increase in weight loss with an increase in temperature. Arrakhiz et al. [37] studied the mechanical and thermal characteristics of Doum/low-density polyethene composites reinforced with natural fibres. It was discovered that there had been a 30% decrease in weight loss overall. The thermal and mechanical characteristics of composites reinforced with bamboo fibre were studied by Chin et al. [38]. At weight losses of 5 and 50%, they measured the BFRC thermal breakdown temperature. In our investigation, a similar outcome (a weight drop of almost 50%) was seen.

### 3.2. Heat Deflection Temperature

**Figure 8:**
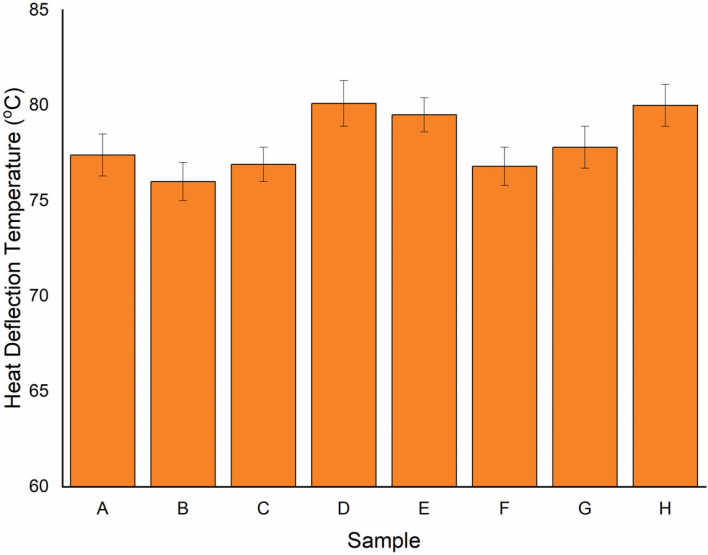
Heat deflection temperature for various samples.

Another important parameter for analysis of the thermal behaviour of the material is heat deflection temperature. The material’s stiffness is measured with time and temperature changes. It is a way to evaluate how much strain a polymer can take before breaking down at high temperatures. Figure 8 shows the heat deflection temperature of various samples. The heat deflection was highest in sample D. This may be due to the combination of RRFRR, wherein flax fibre was placed between the ramie fibre. This layer of ramie fibre is a barrier against heat, which could be very beneficial for the material and thus help improve the heat deflection temperature. The lowest heat deflection temperature was in sample B. A difference of 5% was observed between the highest and the lowest heat deflection temperature. Samples D and H had almost the same heat deflection temperature. However, samples A and G had a 3% difference, followed by samples C and F with 4%, and E was found to be 1%. The effects of heat treatment on the thermal and mechanical properties of ramie-fabric-reinforced polylactic acid biocomposites were investigated by Chen et al. [39], and it was observed that the fibre composite with heat treatment showed a better heat deflection temperature as compared to the without-heat-treatment composite. Ramesh et al. [40] found a highest heat deflection temperature of 103.4 °C from the thermal analysis of a Kevlar/basalt-reinforced hybrid polymer composite. Reddy et al. [41] investigated the heat deflection and thermal conductivity of basalt-fibre-reinforced composites prepared using the hand layup method. They found a highest heat deflection temperature of 95 °C. The present study found the maximum heat deflection temperature to be 80 °C. Hence, with the above results, we can attribute the lower heat deflection temperature in the present study to all the samples. However, comparing the above result (without heat treatment) with the present investigation, it was found that the current results are slightly better. This is because the combination of ramie fibre and flax fibre showed a better heat deflection temperature.

### 3.3. Coefficient of Thermal Expansion

**Figure 9:**
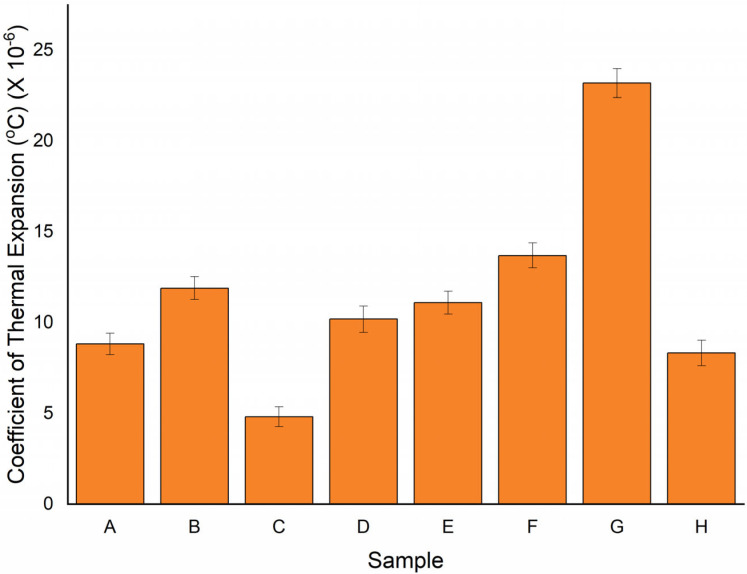
Coefficient of thermal expansion for various samples.

Figure 9 shows the coefficient of thermal expansion of various samples. The rate at which a material expands when heated is measured in terms of its coefficient of thermal expansion. The coefficient of thermal expansion was found to be higher in sample G. The sample exhibited the FRRRF combination. This combination of the sample resembles the weak bonding strength of the resin with the fibre. However, the lowest coefficient of thermal expansion was found in the C sample, which showed a better bonding strength of the resin with the fibre. Mittal et al. [42] reported during their investigation that heat transfer occurred isotopically within the material. It occurs due to the cross-ply weaving of the reinforced fibre. The lowest coefficient of thermal expansion was found in the C sample. The maximum increase in the coefficient of thermal expansion was found to be 380%. However, the minimum difference (82%) was observed in samples A and H. From the above study, we can conclude that the sample with three layers of ramie is unsuitable as it has a high coefficient of thermal expansion. Van et al. [43] investigated several fibre composite properties using thermoplastic polymers. The use of natural fibre was observed to be better than the thermoplastic polymer, and the result showed a low coefficient of linear thermal expansion in the present investigation (using natural fibre). Saidane et al. [44] investigated the thermomechanical behaviour of flax/green epoxy composites. Internal stress was calculated using thermal expansion coefficients, and it was observed that the coefficient of thermal expansion for the natural flax fibre was in the range of 1.23 × 10^−6^/°C to 1.77 × 10^−6^/°C. A similar range was observed in the present study for the developed hybrid composite. Improvements in the thermal behaviour of date-palm/bamboo-fibre-reinforced epoxy hybrid composites were studied by Jawaid et al. [45]. They found that the coefficient of thermal expansion was higher in the date palm fruit bunch stalk/bamboo (AA/B) and lower in the date palm fruit leaf stalk/bamboo (A/B) at 135.7 µm/m °C.

### 3.4. Thermal Conductivity

**Figure 10:**
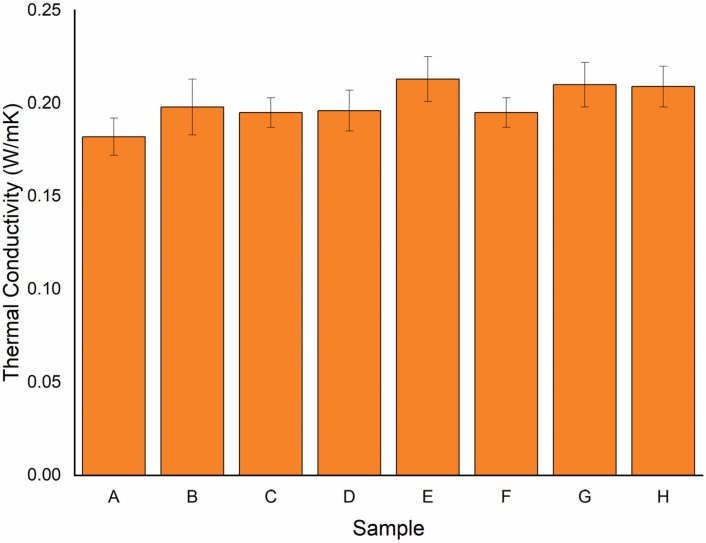
Graph showing thermal conductivity for various samples.

Figure 10 shows the thermal conductivity of the samples. *Thermal conductivity* describes the ability of a material to conduct or transfer heat. In this test, it was observed that the highest thermal conductivity was found in sample E, which was observed to be 0.213 W/mK. The lowest conductive sample was found to be sample A, with a thermal conductivity of 0.182 W/mK. The percentage drop was observed to be 15%. However, the present study samples with the same consecutive layers (samples G and H) at the centre were more suitable. They designate a meagre difference in thermal conductivity (i.e., 1% and 2% drop) and are more stable than the remaining samples. Anand et al. [46] reported a maximum increase in thermal conductivity of 0.292 W/mK, investigated with the combination of Kevlar and basalt fibre composite. The increase in thermal conductivity was approximately 21%. Li et al., investigated thermal diffusivity, thermal conductivity, and specific heat of flax fibre–HDPE biocomposites at processing temperatures. The thermal conductivity was observed from 0.4018 W/mK to 0.3367 W/mK. However, in the present study, the lower thermal conductivity may be due to the high fibre content on the composite. It is well documented in the literature that the increase in fibre reduces the thermal conductivity of the material [47]. Sharma et al. [48] conducted an experimental investigation into the mechanical and thermal properties of a needle-punched nonwoven jute fibre/epoxy composite filled with marble dust particles. They noticed that adding filler increased thermal conductivity (0.29 to 0.38 using marble dust). Another investigation by Soleimani et al. [49] showed the result of fibre pretreatment and compatibilizer on the mechanical and material properties of flax fibre–polypropylene biocomposites. The thermal conductivity was found to be in the range of 0.126 W/mK to 0.152 W/mK. It was observed that adding ramie fibre to the matrix increased the material’s thermal conductivity to a greater extent, as given in Table 3.

**Figure 11:**
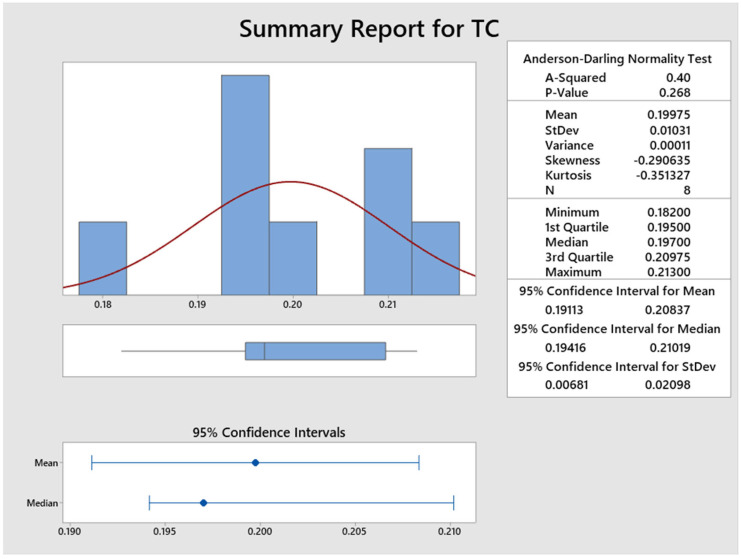
Graph showing statistical analysis of thermal conductivity.

**Figure 12:**
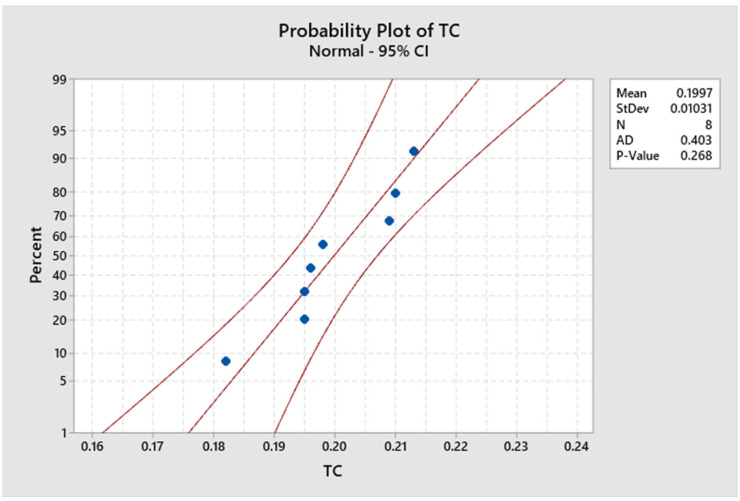
Graph showing statistical analysis of thermal conductivity.

Figure 11 and Figure 12 illustrate the statistical analysis of response thermal conductivity values acquired using the Minitab 19 software presented above. It is identified that the probability of the experimental value of thermal conductivity is 0.268 since *p*-values are greater than 0.05, confirming that it is normally distributed, and that the experimental data are accepted. From Figure 12, for 95% of the significance level, similar *p*-values are obtained. It is observed that the minimum and maximum values are 0.182 to 0.213 (W/mK).

### 3.5. Morphological Analysis

**Figure 13:**
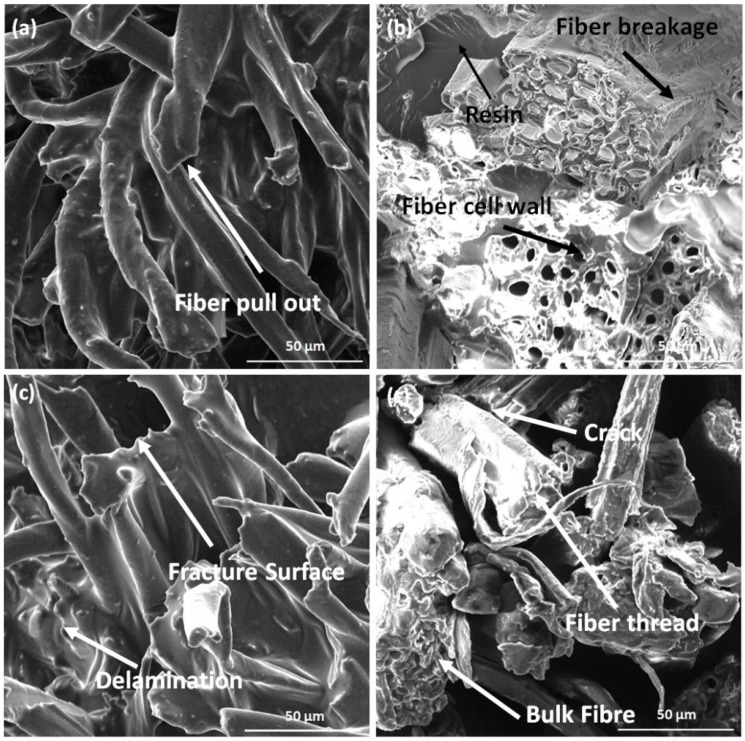
SEM micrograph of (**a**) sample B, (**b**) sample D, (**c**) sample G, and (**d**) sample H.

Microstructural analysis was conducted using a scanning electron microscope (SEM- JOEL JSM 6490). The surface morphology was studied after the heat deflection temperature test. Figure 13a–d shows the micrograph of various samples (B, D, G, and H). All of the sample revealed no voids in the microstructure, which may result in a higher strength of the hybrid composite. Figure 13a shows the failure of the sample due to fibre pull and illustrates the fibre’s dense packing. It can be seen that the ramie fibres are considerable in size and are positioned fairly apart from each other. These natural fibres tend to undergo brittle fractures. Figure 13b shows the fibre cell wall and resin in its microstructure. The bulk fibre in the sample shows a brittle failure with fibre breakage. As the fibres get pulled out the matrix element were observed to retain its shape. This facilitates their ability to absorb the minimal force without the fibres. Similar results were observed by Hamad et al. [50]. Figure 13c shows the failure of the sample due to delamination. A wavy appearance in the micrograph can be observed, representing the failure due to delamination. In addition, the fracture surface of the fibre can be seen in the test. It may be due to the tear of the fibre. Flax fibres are considerably thin compared to ramie fibres. The flax fibres are also closer to each other. This facilitates superior bonding with the matrix element. The combined influence of the fibre and matrix with good adhesion enables the hybrid laminate to absorb greater load. Figure 13d shows the failure due to the crack. In the present sample, the pull-out failure of the fibre can be seen, followed by a crack. The crack may be formed due to improperly binding epoxy/resin with fibre. In the absence of reinforcement fibres, the matrix element gets crumbled and wither due to the excess load. However, along with the fibres, the matrix element is much more capable of absorbing the external load.

## 4. Conclusions

In this study, the thermal analysis of the sample was analysed by varying the stacking sequence of the ramie and flax fibre. The following conclusions were drawn from the sample’s thermal analysis:

1. TGA analysis showed three stages of weight loss (%). The sample showed less weight loss during the first stage, followed by the second and third stages.
2. At the third stage, the sample weight was reduced drastically for all of the samples, which was due to the degradation of fibres and hydrogen elimination.
3. The maximum temperature was observed for all the samples at 630 °C.
4. The heat deflection temperature was highest in sample D (80.1 °C) and lowest in sample B (76.0 °C). The outer layer of ramie fibre with a single flax fibre showed better results than other samples. This is because the resin bonds well with the RRFRR hybrid structure and, thus, provides better resistance to heat deflection.
5. The coefficient of thermal expansion was found to be low in sample C. On the other hand, a high coefficient of thermal expansion was observed in sample G. Flax fibre at the outer part of the sample has shown markable resistance towards thermal expansion, which makes it suitable for nonuniform changes in the dimension at higher temperatures and provides more stability to the material.
6. The highest thermal conductivity was found in sample E, which was observed to be 0.213 W/mK. Conversely, the lowest conductive sample was found for sample A, i.e., 0.182 W/mK.

This study is limited in that it considered only thermal properties to evaluate the hybrid composites. However, it should be noted that this article is a continuation of a previous work, where, by analysing the mechanical properties, it was shown that laminate H outperforms other laminate designs.

## Figures and Tables

**Table 1:** Physical properties of the reinforcement materials.

Physical Properties	Flax Fibre	Ramie Fibre	Phenol–Formaldehyde Resin
Density (g/cm^3^)	1.50	1.56	1.3
Tensile strength (MPa)	800	1000	3800
Young’s modulus (GPa)	27.6	61.4–128	
Elongation to break (%)	2.7–3.2	3.6–3.8	2
Thermal conductivity (W/mK)			0.25

**Table 2:** Stacking sequences of the hybrid composites show the composite volume and thicknesses.

Sample	Specimen	Volume of Laminate	Thickness of Flax Fibre (mm)	Thickness of Ramie Fibre (mm)
A	FFFFF	417,600	4.15	-
B	RRRRR	487,800	-	4.60
C	FFRFF	458,100	3.32	0.92
D	RRFRR	441,900	0.83	3.68
E	FRFRF	432,900	2.49	1.84
F	RFRFR	416,700	1.66	2.76
G	FRRRF	477,900	1.66	2.76
H	RFFFR	461,700	2.49	1.84

F—Flax, R—Ramie.

**Table 3:** Experimental result of composite.

Sample	Specimen	Heat Deflection Temperature (°C)	Coefficient of Thermal Expansion (°C)	Thermal Conductivity (W/mK)
A	FFFFF	77.4	8.83 × 10^−6^	0.182
B	RRRRR	76.0	1.19 × 10^−5^	0.198
C	FFRFF	76.9	4.83 × 10^−6^	0.195
D	RRFRR	80.1	1.02 × 10^−5^	0.196
E	FRFRF	79.5	1.11 × 10^−5^	0.213
F	RFRFR	76.8	1.37 × 10^−5^	0.195
G	FRRRF	77.8	2.32 × 10^−5^	0.21
H	RFFFR	80.0	8.83 × 10^−6^	0.209

F—Flax, R—Ramie.

## Data Availability

The data presented in this study are available through email upon request to the corresponding author.
